# Respiratory and cardiovascular responses to walking down a traffic-polluted road compared with walking in a traffic-free area in participants aged 60 years and older with chronic lung or heart disease and age-matched healthy controls: a randomised, crossover study

**DOI:** 10.1016/S0140-6736(17)32643-0

**Published:** 2018-01-27

**Authors:** Rudy Sinharay, Jicheng Gong, Benjamin Barratt, Pamela Ohman-Strickland, Sabine Ernst, Frank J Kelly, Junfeng (Jim) Zhang, Peter Collins, Paul Cullinan, Kian Fan Chung

**Affiliations:** a National Heart and Lung Institute and MRC-PHE Centre for Environment and Health, Imperial College, London, UK; b NIHR Biomedical Research Unit, Royal Brompton & Harefield NHS Trust, London, UK; c BIC-ESAT and SKL-ESPC, College of Environmental Sciences and Engineering, Peking University, Beijing China; d Nicholas School of Environment & Duke Global Health Institute, Duke University, Durham, USA; e Duke Kunshan University, Kunshan, China; f MRC-PHE Centre for Environment and Health, King's College London, London, UK; g Rutgers School of Public Health, Rutgers, The State University of New Jersey, New Jersey, USA

## Abstract

**Background:**

Long-term exposure to pollution can lead to an increase in the rate of decline of lung function, especially in older individuals and in those with chronic obstructive pulmonary disease (COPD), whereas shorter-term exposure at higher pollution levels has been implicated in causing excess deaths from ischaemic heart disease and exacerbations of COPD. We aimed to assess the effects on respiratory and cardiovascular responses of walking down a busy street with high levels of pollution compared with walking in a traffic-free area with lower pollution levels in older adults.

**Methods:**

In this randomised, crossover study, we recruited men and women aged 60 years and older with angiographically proven stable ischaemic heart disease or stage 2 Global initiative for Obstructive Lung Disease (GOLD) COPD who had been clinically stable for 6 months, and age-matched healthy volunteers. Individuals with ischaemic heart disease or COPD were recruited from existing databases or outpatient respiratory and cardiology clinics at the Royal Brompton & Harefield NHS Foundation Trust and age-matched healthy volunteers using advertising and existing databases. All participants had abstained from smoking for at least 12 months and medications were taken as recommended by participants' doctors during the study. Participants were randomly assigned by drawing numbered disks at random from a bag to do a 2 h walk either along a commercial street in London (Oxford Street) or in an urban park (Hyde Park). Baseline measurements of participants were taken before the walk in the hospital laboratory. During each walk session, black carbon, particulate matter (PM) concentrations, ultrafine particles, and nitrogen dioxide (NO_2_) concentrations were measured.

**Findings:**

Between October, 2012, and June, 2014, we screened 135 participants, of whom 40 healthy volunteers, 40 individuals with COPD, and 39 with ischaemic heart disease were recruited. Concentrations of black carbon, NO_2_, PM_10_, PM_2.5_, and ultrafine particles were higher on Oxford Street than in Hyde Park. Participants with COPD reported more cough (odds ratio [OR] 1·95, 95% CI 0·96–3·95; p<0·1), sputum (3·15, 1·39–7·13; p<0·05), shortness of breath (1·86, 0·97–3·57; p<0·1), and wheeze (4·00, 1·52–10·50; p<0·05) after walking down Oxford Street compared with Hyde Park. In all participants, irrespective of their disease status, walking in Hyde Park led to an increase in lung function (forced expiratory volume in the first second [FEV_1_] and forced vital capacity [FVC]) and a decrease in pulse wave velocity (PWV) and augmentation index up to 26 h after the walk. By contrast, these beneficial responses were attenuated after walking on Oxford Street. In participants with COPD, a reduction in FEV_1_ and FVC, and an increase in R5–20 were associated with an increase in during-walk exposure to NO_2_, ultrafine particles and PM_2.5_, and an increase in PWV and augmentation index with NO_2_ and ultrafine particles. In healthy volunteers, PWV and augmentation index were associated both with black carbon and ultrafine particles.

**Interpretation:**

Short-term exposure to traffic pollution prevents the beneficial cardiopulmonary effects of walking in people with COPD, ischaemic heart disease, and those free from chronic cardiopulmonary diseases. Medication use might reduce the adverse effects of air pollution in individuals with ischaemic heart disease. Policies should aim to control ambient levels of air pollution along busy streets in view of these negative health effects.

**Funding:**

British Heart Foundation.

**Research in context**

**Evidence before study**

We searched PubMed without language restriction for reports published up to July, 2017, using the following search terms: “air pollution”, “particulate matter”, “diesel exhaust”, “exercise”, “walking”, and “physical activity”, in addition to the terms “COPD” and “ischaemic heart disease”. We found no studies that have examined the effect of walking in a polluted area on cardiovascular and respiratory responses. Most of the reports analysed the effect of submaximal exercise, usually cycling or running, in relatively young volunteers. No studies looked at the effect of walking at a normal pace, apart from our own study in a group of asthmatic participants (McCreanor et al, 2007). The effect of exercising in participants with chronic obstructive pulmonary disease (COPD) or ischaemic heart disease in polluted areas has not been studied, apart from one study by Superko et al (1984) that looked at COPD participants during brisk walking on a treadmill.

**Added value of study**

Our findings show for the first time the detrimental effect of air pollution on walking at a normal pace. We document the beneficial cardiorespiratory benefits of walking in healthy volunteers aged 60 years and older, an effect that is lost when walking in a polluted environment. Our results show that this similar phenomenon is happening in participants with COPD and ischaemic heart disease, although the cardiac medication taken by the ischaemic heart disease participants might be protecting them against the detrimental cardiovascular effects of air pollution.

**Implications of all the available evidence**

Our findings suggest that healthy people, as well as those with chronic cardiorespiratory disorders, should minimise walking on streets with high levels of pollution because this curtails or even reverses the cardiorespiratory benefits of exercise. Instead, walking exercise should be enjoyed in urban green space areas away from high density traffic. Patients with chronic cardiorespiratory disorders should be encouraged to use appropriate medication to mitigate the adverse effects of air pollution. Current ambient levels of air pollution along busy streets are unacceptable and need to be controlled.

## Introduction

Air pollution currently represents one of the world's most important environmental health risks and has been associated with an increase in premature deaths, mostly caused by ischaemic heart disease and chronic obstructive pulmonary disease (COPD).^1^ Air pollution caused by emissions from diesel-powered vehicles and from tyres and brakes of motor vehicles comprises of particulate matter (PM) such as PM_10_, PM_2.5_, and ultrafine particles (PM with a diameter ≤10 μm, 2·5 μm and 0·1 μm, respectively) as as noxious gases such as nitrogen dioxide (NO_2_). These individual pollutants or their mixture have been associated with increasing early deaths and deleterious respiratory and cardiovascular events.^2^, ^3^, ^4^, ^5^ Long-term exposure to pollution over years can lead to an increase in the rate of decline of lung function in non-smoking adults, particularly in the elderly^6^, ^7^ and in individuals with COPD.^8^, ^9^ Short term exposure over days to higher pollution levels has been associated with increased exacerbations and hospital admissions with a reduction in lung function in patients with COPD^10^, ^11^, ^12^and has also been implicated in triggering acute episodes of cardiac ischaemia and in causing excess deaths from ischaemic heart disease.^13^

Exposure of healthy exercising individuals to reconstituted diesel exhaust particles causes an acute bronchoconstrictor response with increased arterial stiffness and pulmonary inflammation.^14^, ^15^, ^16^, ^17^ Indeed, findings of studies have shown that exposure to diesel exhaust causes an immediate and transient increase in arterial stiffness in healthy volunteers.^14^, ^18^ Further, acute myocardial ischaemia measured by ST-segment depression has been reported in individuals with ischaemic heart disease exposed to diesel exhaust particles in exposure chambers.^19^ In one study,^20^ exposure of individuals with asthma to high levels of diesel vehicle pollution while walking on a busy commercial street (Oxford Street) in London, UK, led to a fall in forced expiratory volume in the first second (FEV_1_) and pulmonary inflammation.

Because of the paucity of information on the effect of air pollution on participants with COPD and ischaemic heart disease, we aimed to assess the effects on respiratory and cardiovascular responses of a short-term exposure to traffic pollution during a 2-h walk in a busy London street compared with those in a nearby park with lower pollution levels.

## Methods

### Study design and participants

In this randomised, crossover study, we recruited healthy volunteers using advertising in public areas within the Royal Brompton Hospital, and individuals with COPD or ischaemic heart disease from existing databases or outpatient respiratory and cardiology clinics at the Royal Brompton & Harefield NHS Foundation Trust (table). To be eligible, individuals with COPD had to be aged 60 years and older with Global initiative for Obstructive Lung Disease (GOLD) stage 2 disease with an FEV_1_ to forced vital capacity (FVC) ratio of less than 0·7 and a predicted FEV_1_ of 80% or less. Individuals with ischaemic heart disease had to have angiographic evidence of ischaemic heart disease. Healthy volunteers and participants with ischaemic heart disease had to show no evidence of airflow obstruction. All participants had abstained from smoking for at least 12 months. Patients with COPD or ischaemic heart disease had been clinically stable for 6 months, without any deterioration in symptoms or episodes of angina for participants with ischaemic heart disease. Medications were taken as recommended by participants' doctors during the study.

**Table:** Baseline characteristics of participants

		**Healthy (n=40)**	**COPD (n=40)**	**IHD (n=39)**
Sex				
	Male	19 (48%)	19 (48%)	35 (90%)
	Female	21 (53%)	21 (53%)	4 (10%)
Age (years)		61·8 (1·2)	67·6 (1·1)*	66·9 (1·4)*
Smoking history (pack-years)		3·9 (1·50)	36·7 (4·50)*	9·0 (2·4)
Ex-smokers		14 (35%)	37 (93%)	25 (64%)
Never smokers		26 (65%)	3 (8%)	14 (36%)
MRC breathlessness scale		0·1 (0·1)	1·2 (0·1)*	0·7 (0·1)*
Cough score		0·03 (0·03)	0·33 (0·10)*	0·13 (0·07)
Sputum score		0·03 (0·03)	0·35 (0·12)*	0·11 (0·05)
Shortness of breath score		0	0·2 (0·09)*	0·03 (0·03)
Wheeze score		0	0·15 (0·08)	0
FEV_1_ (L)		2·78 (0·59)	1·50 (0·44)*	2·62 (0·72)
FEV_1_ (% predicted)		96·1 (2·1)	57·9 (2·2)*	94·8 (2·8)
FVC (L)		3·88 (0·95)	2·98 (1·01)*	3·50 (0·94)
FVC (% predicted)		104 (2·4)	88·4 (3·1)*	99·8 (3·9)
Impulse oscillation 5 Hz (kPa/L/s)		0·42 (0·03)	0·58 (0·03)*	0·45 (0·02)
Impulse oscillation 20 Hz (kPa/L/s)		0·35 (0·02)	0·38 (0·02)	0·36 (0·02)
Pulse wave velocity (m/s)		8·39 (0·27)	9·59 (0·27)*	9·49* (0·34)
Augmentation index (%)		26·24 (1·03)	25·29 (1·66)	20·44 (2·18)*
FeNO (ppb)		26·3 (3·7)	37·6 (4·8)	29·6 (5·2)
Medication				
	Any oral medication	16 (40%)	24 (60%)	38 (97%)
	β blocker	1 (3%)	0	28 (72%)
	ACEI	2 (5%)	9 (23%)	26 (67%)
	ARB	0	2 (5%)	9 (23%)
	Aspirin	2 (5%)	3 (8%)	32 (87%)
	Clopidogrel	0	0	12 (31%)
	Oral nitrate	0	0	8 (21%)
	Glyceryl trinitrate sublingual, as needed	0	0	2 (5%)
	Statin	5 (13%)	8 (20%)	31 (80%)
	Calcium-channel blocker	2 (5%)	5 (13%)	9 (23%)
	Diuretic	1 (3%)	2 (5%)	4 (10%)
	Any inhaled medication	0	35 (88%)	0
	Short-acting β-agonist	0	28 (70%)	0
	Long-acting β-agonist or inhaled corticosteroids	0	22 (55%)	0
	Inhaled corticosteroids	0	3 (8%)	0
	Long-acting muscarinic antagonist	0	24 (60%)	0
	Long-acting β-agonist	0	1 (3%)	0

Data are n (%), mean (SD) or n. COPD=chronic obstructive pulmonary disease. IHD=ischaemic heart disease. MRC=Medical Research Council. FEV_1_=forced expiratory volume. FVC=forced vital capacity. FeNO=fractional exhaled nitric oxide. ACEI=angiotensin-converting enzyme inhibitor. ARB=angiotensin II receptor blocker. ppb=parts per billion.

* p<0·05 compared with healthy group.

The experimental sites in London were the western end of Oxford Street where traffic is restricted to buses and taxicabs, most of which are powered by diesel, and the nearby, traffic-free part of Hyde Park.^20^ Participants were randomly assigned to start on either site by drawing numbered disks at random from a bag on the day before the first walk, with participants informed of the starting site only on the day of the walk. Participants were transported to each site from the hospital laboratory 3 km away in a hybrid electric-powered car. Participants walked from 11 am to 1 pm at their own pace, covering an average distance of 5 km on both sites. The walks were separated by 3–8 weeks.

The study was approved by the UK National Research Ethics Service (London City Road and Hampstead Ethics Committee; Research Ethics Number 12/LO/1064). Informed written consent was obtained from all participants.

### Health outcome measures

**Figure 1:**
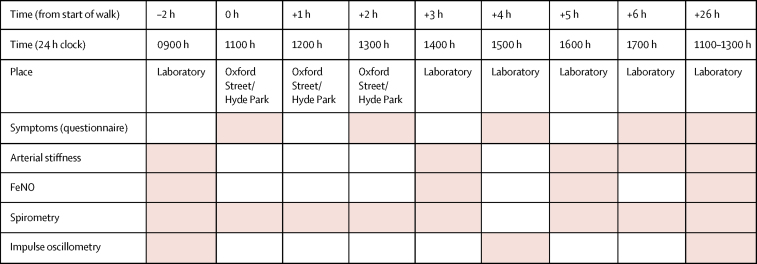
Time course of health outcomes measurements Pink shading indicates timing of measurements.

Baseline measurements were taken before the walk in the hospital laboratory of the Royal Brompton & Harefield NHS Foundation Trust. Figure 1 shows the time course of health outcome measurements. Shortness of breath was measured using a modified Medical Research Council dyspnea scale, while cough, sputum, wheeze, and sweat were scored on a 0 to 4 scale. FEV_1_ and FVC were measured using a spirometer (Vitalograph, Buckingham, UK) and resistance of the respiratory tract at 5 and 20 Hz (R5 and R20) using impulse oscillometry (Master Screen Spirometry-IOS System, Jaeger, Germany).^21^ Fractional exhaled nitric oxide (FeNO) was assessed using a portable electrochemical sensor (NOBreath Bedfont Scientific Ltd, Kent, UK), and pulse wave velocity and augmentation index using a Vicorder (Skidmore Medical, Bristol, UK). Central aortic pulse wave velocity was defined as the ratio of pulse wave transit time and distance between the carotid and femoral arteries while augmentation index was the enhancement of the central aortic pressure by a reflected pulse wave, both being measures of arterial stiffness.

### Exposure measures

During each walk session, PM_2.5_ and PM_10_ concentrations were measured using a light scattering sensor (AM510 SidePak Personal Aerosol Monitors, TSI Ltd, MI, USA). Ultrafine particles number concentrations were measured using a unipolar diffusion charger (Philips Aerosense NanoTracer; size range of 10–300 nm); and black carbon using an optical absorption method (microAeth Model AE51 Black Carbon aerosol monitor; AEthlabs, CA, USA; flow rate 100 mL per min). Temperature and relative humidity were electronically logged, as were noise levels (Bruel and Kjaer Type 2236 Sound level meter, Naerum, Denmark). NO_2_ concentrations were taken from a stationary monitoring site on Oxford Street repeatedly passed during walks on Oxford Street. Because no monitoring was available in Hyde Park, NO_2_ concentrations were taken from the nearest representative location sited in a school playground.

### Statistical analysis

Power calculation was based on the augmentation index as a primary outcome, using previously-published data.^14^ We used two-sided two-group *t* tests conducted at 5% significance to compare the effect of exposure for all participants with disease (n=80) versus control participants (n=40) or participants with either COPD or ischaemic heart disease (n=40) versus control participants. The effect of exposure is the difference in response following Oxford Street versus Hyde Park, with the correlation between within-participant measurements being 0·7 and standard deviations the average of those of Lundbäck and colleagues.^14^ The minimum effect size of the augmentation index is detectable with at least 90% power. The detectable effect size calculated as the minimum detectable difference between the two groups divided by the mean of the control group shows that the group with disease is 59% and 69% different from that of the control group for the 80 versus 40, and for the 40 versus 40 comparisons, respectively, which represents a moderate size effect.

We applied hierarchical proportional odds analyses for the ordinal symptom responses, using PROC GLIMMIX in SAS (version 9.4) to estimate the odds ratios (ORs) of getting worse symptoms. We entered time, group, location, and their interactions to estimate odds ratios, with a random effect to control for repeated measures within participants. We controlled the pre-exposure status by entering the baseline level as a covariate.

We assessed the effects of exposure on health endpoints, measured as continuous variables, with linear mixed-effects models using PROC MIXED (SAS version 9.4) that included absolute changes from baseline as dependent variables. Participant group (healthy, COPD, and ischaemic heart disease), location (Oxford Street *vs* Hyde Park), and time of measurement were categorical fixed effects. Ambient temperature and relative humidity were controlled as additional covariates. We controlled participant as a random effect to account for the variation of health endpoints between participants. We also introduced a covariance structure to model correlations between repeated measurements taken across time on the same day for each participant. The spatial power covariance structure was selected using Akaike information criterion because of its best fit compared with other covariance structures (eg, compound symmetry, unstructured, and spatial power). The interactions between group, time, and location were included in the models to examine whether the exposure-related changes from baseline (post-session minus pre-session) on health endpoints differed between groups.

We studied associations between during-walk concentrations of each measured pollutant, including noise, and health endpoints, using mixed-effects models. In these models, each health endpoint was the dependent variable; and the 2 h average concentration or level of each pollutant and noise was the independent variable. Random-effects variables and structure were the same as described above on comparing exposure and control sessions. We report mean and 95% CIs of changes in each health endpoint associated with IQR changes in pollutant concentrations or noise levels. We only assessed the associations at the time points (relevant to the start of walk) when a significant site effect was recorded for a particular health endpoint.

We did a sensitivity analysis in ischaemic heart disease participants concerning the effect of medication use. The medication use status was controlled in the model as an interaction with time and group in the location analysis model, and with time and air pollution in the association analysis model. We also did a sensitivity analysis on the smoking history of the study participants. We included the participants' pack-year of smoking (defined as pack of 20 cigarettes smoked multiplied by number of years smoked) as an independent variable in the models to test its modifying effect on the changes between sites.

### Role of the funding source

The funder of the study had no role in study design, data collection, data analysis, data interpretation, or writing of the report. The corresponding author had full access to all the data in the study and had final responsibility for the decision to submit for publication.

## Results

**Figure 2:**
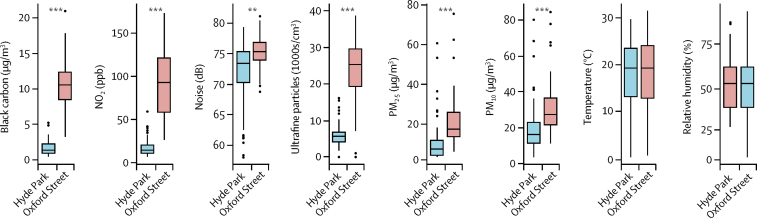
Distribution of black carbon, nitrogen dioxide (NO_2_), noise, ultrafine particles, PM_2.5_ and PM_10_ concentrations, temperature, and relative humidity on the visit days to Oxford Street or to Hyde Park Box plots with 95% CIs. PM=particulate matter. **p<0·01. ***p<0·001.

Between October, 2012, and June, 2014, we screened 135 participants, of whom 40 healthy volunteers, 40 participants with COPD, and 39 with ischaemic heart disease were recruited. Baseline characteristics of participants are shown in the table. Concentrations of black carbon, NO_2_, PM_2.5_, PM_10_, and ultrafine particles and noise level during the walk sessions were all significantly higher on Oxford Street than in Hyde Park (figure 2). There were no significant differences in temperature or relative humidity. The mean distance walked in 2 h was 4·78 km (SD 1·20) for Hyde Park, and 4·62 km (1·01) for Oxford Street.

**Figure 3:**
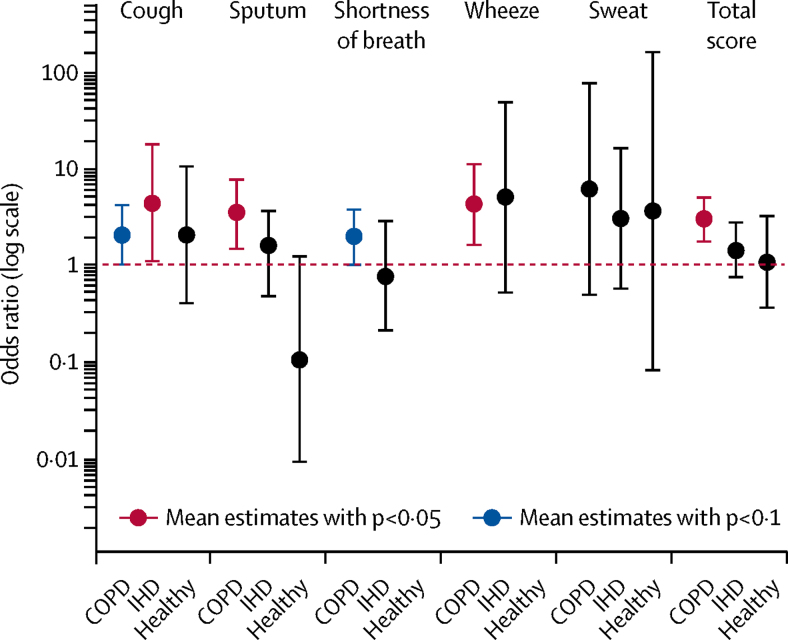
Odds ratio of getting worse symptoms of cough, sputum, shortness of breath, wheeze, sweat, and total scores for all these symptoms at Oxford Street versus Hyde Park for healthy volunteers and participants with COPD or IHD COPD=chronic obstructive pulmonary disease. IHD=ischaemic heart disease.

Participants with COPD reported significantly higher scores on cough (OR 1·95, 95% CI 0·96–3·95; p<0·1), sputum (3·15, 1·39–7·13; p<0·05), shortness of breath (1·86, 0·97–3·57; p<0·1), and wheeze (4·00, 1·52–10·50; p<0·05) after walking down Oxford Street compared with Hyde Park (figure 3). Participants with ischaemic heart disease reported significantly higher scores of cough after the Oxford Street walk versus the Hyde Park walk (OR 4·13, 95% CI 1·05–16·33; p<0·05). Healthy volunteers did not report any increase in symptoms of cough, sputum, shortness of breath, wheeze, or sweat after the Oxford Street walk (figure 3).

**Figure 4:**
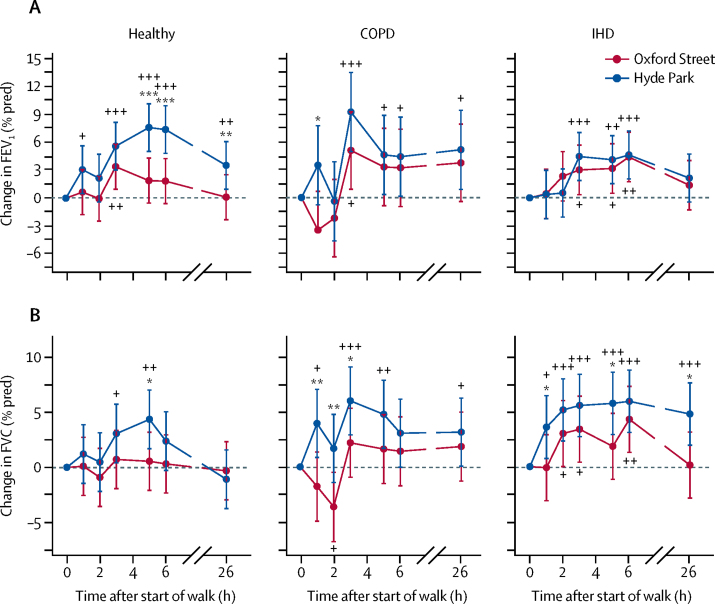
Change in FEV_1_ % of predicted value (A), and FVC % of predicted value (B) from the baseline (time 0) and at 1 and 2 h after the start of the walk in Oxford Street or Hyde Park followed by measurements performed back in the laboratory at times indicated after the start of the walk for healthy volunteers and participants with COPD or IHD Data are percentage changes (95% CI). COPD=chronic obstructive pulmonary disease. IHD=ischaemic heart disease. FEV_1_=forced expiratory volume in the first second. FVC=forced vital capacity. *p<0·05, **p<0·01, ***p<0·001, comparing Oxford Street with Hyde Park. +p<0·05, ++p<0·01, +++p<0·001, compared with timepoint 0.

In healthy volunteers, FEV_1_ increased within 1 h of walking in Hyde Park, maximal at 5 and 6 h with a mean 7·6% rise (95% CI 5·1–10·2); this remained significantly increased by 3·6% (1·0–6·1) at 26 h (figure 4). Participants who walked along Oxford Street only had a small increase in FEV_1_, with a significantly lower improvement compared with walking in Hyde Park (figure 4A; appendix). In participants with COPD and ischaemic heart disease, there were similar increases in FEV_1_ while walking on Hyde Park and Oxford Street at 5 h after the start of the walk, with the increase less than that observed by healthy volunteers in Hyde Park by 3·3% (95% CI −0·9 to 7·5) and 4·6% (0·4 to 8·9) for COPD participants, and by 3·2% (0·5 to 5·8) and 4·1% (1·5 to 6·7) for ischaemic heart disease participants on Oxford Street and Hyde Park, respectively. At the 6 h timepoints, these values remained similar and were 3·2% (−1·0 to 7·4) and 4·4% (0·2 to 8·7) for COPD participants, and 4·4% (1·7 to 7·1) and 4·6% (2·0 to 7·2) for ischaemic heart disease participants on Oxford Street and Hyde Park, respectively. There were no significant differences in FEV_1_ observed between Oxford Street and Hyde Park in either the COPD or ischaemic heart disease group after the end of walk (figure 4A).

In healthy volunteers, there was a significant improvement in FVC at 3 and 5 h after walking in Hyde Park by 3·07% (95% CI 0·41–5·73) and 4·36% (1·70–7·02), respectively; this was not the case after walking on Oxford Street at any timepoint from the baseline (figure 4B). There was an increase in FVC at 1, 3, and 5 h in participants with COPD and at 1, 2, 3, 5, 6, and 26 h in participants with ischaemic heart disease during the Hyde Park walk; these values were higher than those measured during the Oxford Street walk at 1, 2, and 3 h, and 1, 5, and 26 h, respectively (figure 4B).

**Figure 5:**
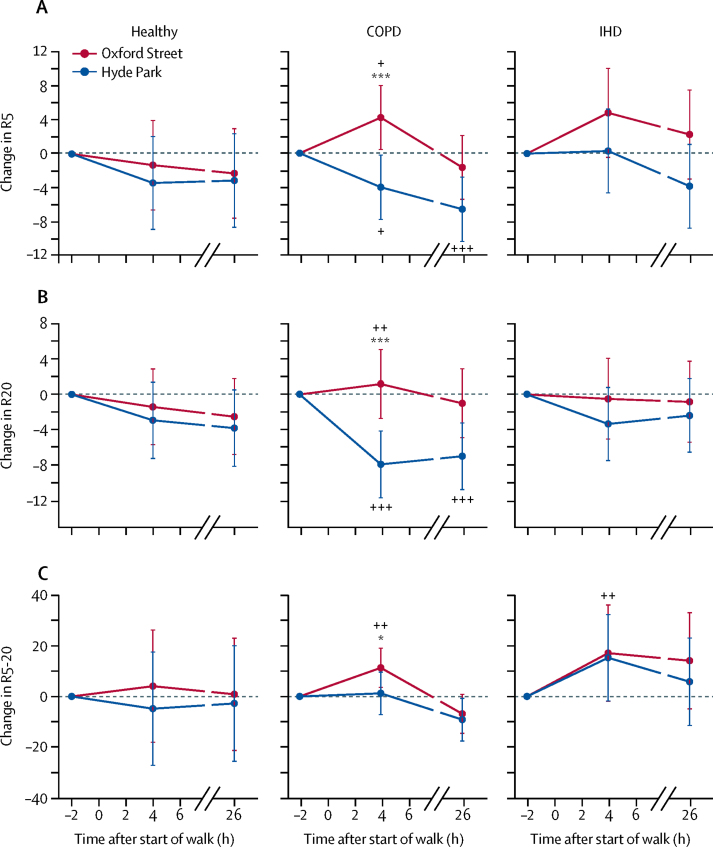
Respiratory resistance at 5 Hz (A; R5) and at 20 Hz (B; R20), and differential frequency-dependent respiratory resistance at 5 Hz and 20 Hz measured by impulse oscillometry (C; R5–20) from baseline (time 0) and at 4 h and 26 h after the start of the walk in Oxford Street or in Hyde Park for healthy volunteers and participants with COPD or IHD Data are percentage changes (95% CI). COPD=chronic obstructive pulmonary disease. IHD=ischaemic heart disease. *p<0·05, **p<0·01, ***p<0·001, comparing Oxford Street with Hyde Park. +p<0·05, ++p<0·01, +++p<0·001, compared with timepoint −2 h.

For the differential frequency-dependent respiratory resistance at 5 Hz and 20 Hz (R5 and R20), there were no significant site differences in changes at 4 or 26 h after walking in healthy volunteers and participants with ischaemic heart disease (figure 5). However, participants with COPD walking on Oxford Street showed a significant increase in R5 and R20 at 4 h compared with walking in Hyde Park. We calculated the differential frequency-dependent respiratory resistance at 5 Hz and 20 Hz (R5–20) as reflecting small airways. R5–20 was significantly increased in participants with COPD at 4 h after the start of the walk on Oxford Street compared with Hyde Park; this difference disappeared by 26 h (figure 5C).

**Figure 6:**
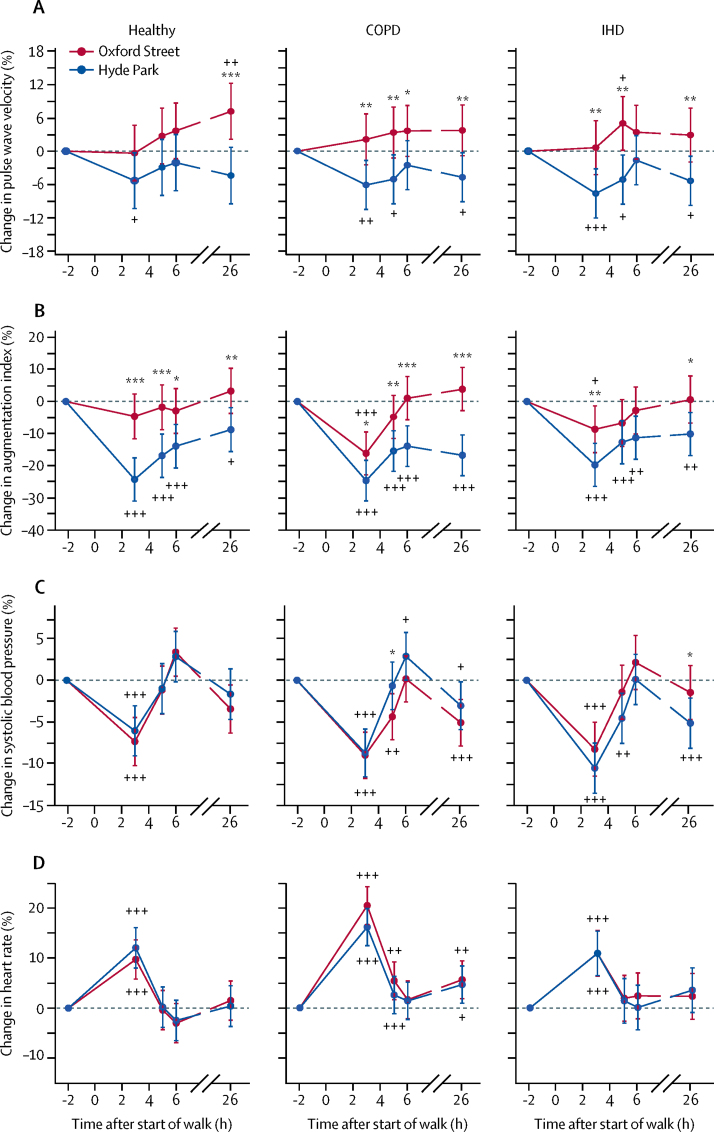
Pulse wave velocity (A), augmentation index (B), systolic blood pressure (C), and in heart rate (D) from the baseline (time 0) and at 1 and 2 h after the start of the walk on Oxford Street or in Hyde Park followed by measurements performed back in the laboratory at times indicated after the start of the walk for healthy volunteers and participants with COPD or IHD Data are percentage changes (95% CI). COPD=chronic obstructive pulmonary disease. IHD=ischaemic heart disease. *p<0·05, **p<0·01, ***p<0·001, comparing Oxford Street with Hyde Park. +p<0·05, ++p<0·01, +++p<0·001, compared with timepoint −2 h.

Pulse wave velocity in healthy volunteers showed a reduction from the baseline by −5·31% (95% CI −10·40 to −0·27) after the Hyde Park walk at time 3 h and an increase from the baseline by 7·17% (2·16–12·20) after the Oxford Street walk at 26 h, with site difference reaching significance at 26 h (figure 6A; appendix; p=0·0007). Pulse wave velocity in participants with COPD or ischaemic heart disease showed similar trends after walks at both sites, but significant site-differences were seen at several timepoints (3, 5, 6, and 26 h for participants with COPD with p<0·04, and 3, 5, and 26 h for ischaemic heart disease with p<0·008).

In healthy volunteers, there was a significant reduction in augmentation index at all timepoints after the Hyde Park walk versus the Oxford Street walk with the largest reduction by −24·27% (95% CI −31·0 to −17·50) at 3 h after the Hyde Park walk; but after Oxford Street, there were no significant changes in augmentation index with the largest reduction by −4·62% (−11·60 to 2·36) at 3 h after the Oxford Street walk (figure 6B; appendix). We noted similar trends in participants with COPD and ischaemic heart disease, but while the decrease in augmentation index after Hyde Park at 3 h was similar to that noted in healthy volunteers, the decrease at 3 h after Hyde Park in participants with COPD and ischaemic heart disease was higher than that reported in healthy volunteers (figure 6). The change in augmentation index after Hyde Park was significantly greater than after Oxford Street at 3, 5, 6, and 26 h for participants with COPD with the largest reduction by −24·64% (95% CI −31·00 to −18·30) at 3 h after Hyde Park versus −16·16% (−22·90 to −9·47) after Oxford Street. For ischaemic heart patients, the largest reduction was by −19·75% (−26·50 to −13·00) at 3 h after Hyde Park versus −8·64% (−15·90 to −1·35) after Oxford Street (figure 6). Therefore, similar to the pulse wave velocity results, walking in Hyde Park led to a reduction (ie, an improvement) in augmentation index for all groups.

In all groups, we noted significant decreases in systolic blood pressure and increases in heart rate at 3 h from baseline, but there were no significant site differences apart from systolic blood pressure being lower at 5 h in COPD after walking down Oxford Street and higher at 26 h in participants with ischaemic heart disease after Oxford Street, compared with after the Hyde Park walk (figure 6C, D). We recorded no significant differences in diastolic blood pressure after walks in Hyde Park or Oxford Street in the three groups.

**Figure 7:**
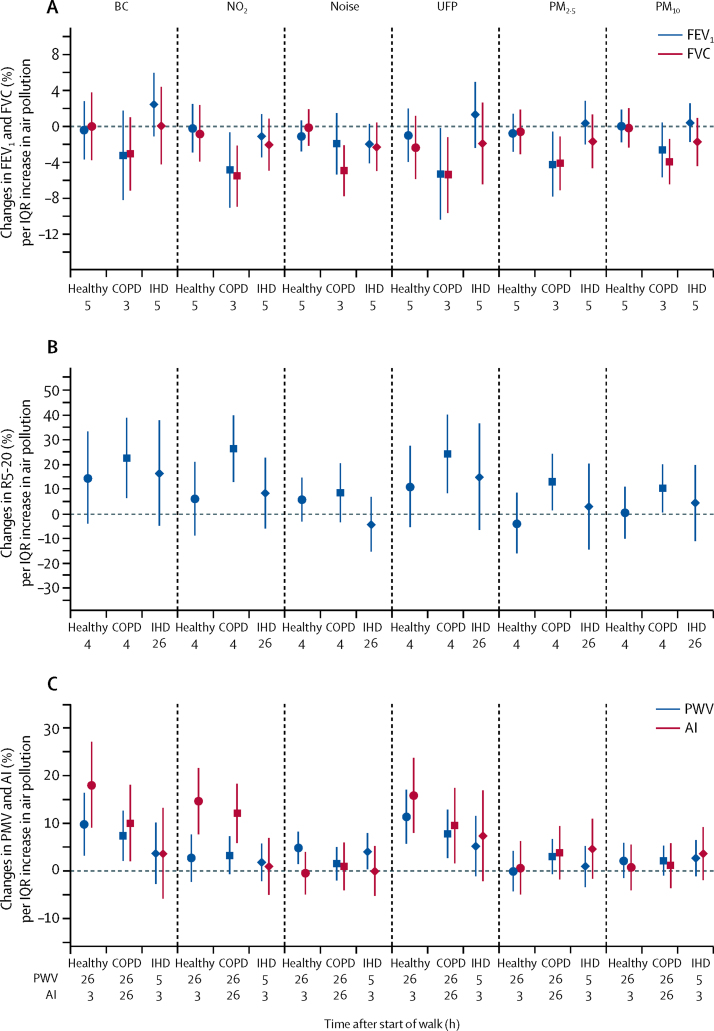
Changes in FEV_1_ (% predicted value) and FVC (% predicted value; A), in differential frequency-dependent respiratory resistance at 5Hz and 20Hz (R5-20, B) and in pulse wave velocity and augmentation index (C) per IQR changes in components of air pollution at the times indicated after the start of the 2 h walk in two exposure sessions for healthy volunteers and participants with COPD or IHD Data are percentage changes (95% CI). BC=black carbon (per 9·2 μg/m^3^). NO_2_=nitrogen dioxide (per 64·9 ppb). UFP=ultrafine particle number concentration (per 19 854/cm^3^). PM_2.5_=particles <2·5 μM in diameter (per 14·94 μg/m^3^). PM_10_=particles <10 μM in diameter (per 14·47 μg/m^3^). FEV_1_=forced expiratory volume in the first second. FVC=forced vital capacity. COPD=chronic obstructive pulmonary disease. IHD=ischaemic heart disease. PWV=pulse wave velocity. AI=augmentation index. Noise per 3·97 dB.

In participants with COPD, a reduction in FEV_1_ and FVC at 3 h was significantly associated with increasing PM_2.5_, ultrafine particles, and NO_2_. A reduction in FVC was also significantly associated with increasing PM_10_ concentrations and noise levels (appendix). An increase in R5–20 at 4 h in participants with COPD was significantly associated with increasing PM_2.5_, black carbon, ultrafine particles and, NO_2_, and PM_10_ (appendix). By contrast, no significant associations for lung function parameters were recorded in healthy volunteers or participants with ischaemic heart disease (figure 7A, B).

In terms of associations between pollutant and noise and arterial stiffness parameters, an increase in pulse wave velocity was significantly associated with increasing black carbon and ultrafine particles in both healthy volunteers and individuals with COPD and with increasing noise in both healthy volunteers and those with ischaemic heart disease (appendix). We also found that an increase in augmentation index was significantly associated with increasing black carbon, ultrafine particles, and NO_2_ in both healthy volunteers and COPD but not in participants with ischaemic heart disease (figure 7C).

**Figure 8:**
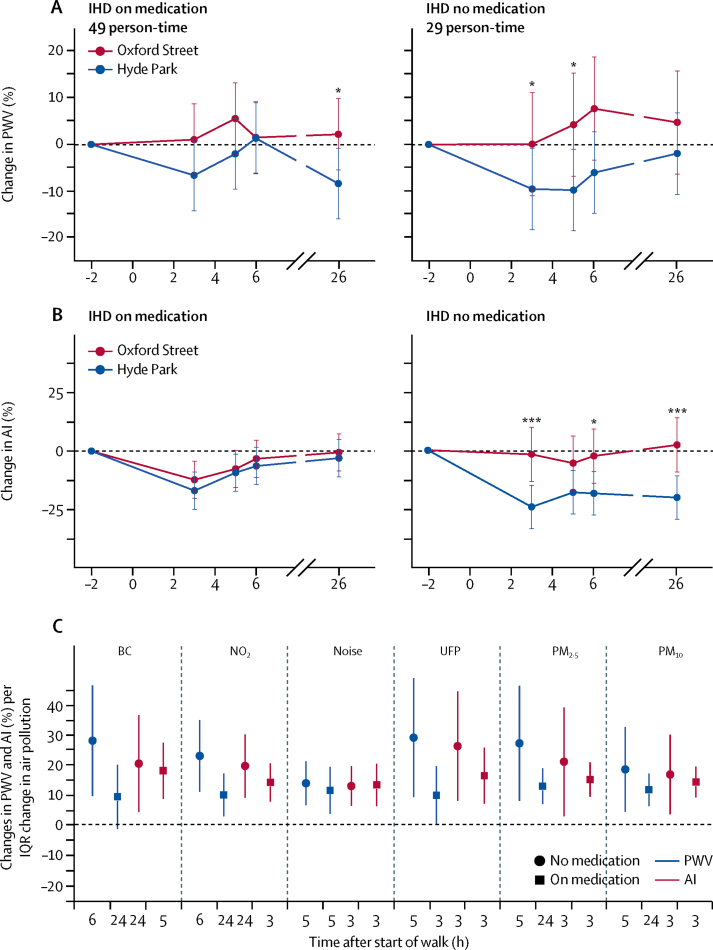
Effect of medication on cardiovascular responses in participants with IHD (A) and (B) show the responses in PWV and AI in participants with IHD stratified according to taking or not taking cardiovascular medication. (C) Shows the sensitivity analysis of the association between PWV and AI per IQR changes in components of air pollution at the times indicated after the start of the 2 h walk in two exposure sessions in the two subgroups of IHD participants. In the 78 walks of the 39 IHD participants, 49 walks were done with participants taking oral medication on that day, with the remaining 29 walks with participants not using medication. IHD=ischaemic heart disease. PWV=pulse wave velocity. AI=augmentation index. BC=black carbon (per 9·2 μg/m^3^). NO_2_=nitrogen dioxide (per 64·9 ppb). UFP=ultrafine particle number concentration (per 19 854/cm^3^). PM_2.5_=particles <2·5 μM in diameter (per 14·94 μg/m^3^). PM_10_=particles <10 μM in diameter (per 14·47 μg/m^3^). * p<0·05. ***p<0·001.

Sensitivity analyses of participants with ischaemic heart disease, stratified by medication use versus no medication use, showed that those who did not use oral medications had significantly attenuated reductions in pulse wave velocity and augmentation index after the Oxford Street walk compared with the reductions after the Hyde Park walk (figure 8). By contrast, participants with ischaemic heart disease who used medications did not show significant differences in pulse wave velocity and augmentation index changes after walks down Oxford Street or in Hyde Park.

We also did a sensitivity analysis by introducing an interaction term between medication use (table), site, and timepoints. The site effects on augmentation index were significantly different between the two groups (no medication or on medication) at 3 h and 26 h (p=0·042 and 0·019, respectively), while no significant differences in site effects on pulse wave velocity were recorded. We also tested whether medication modified the location effects on pulse wave velocity and augmentation index, assuming the modification effect was the same across all timepoints, and noted no significant interaction between location and medication for pulse wave velocity (p=0·718), but the interaction of location and medication for augmentation index was significant (p=0·005). The results were consistent with those of the three-way interaction, which allowed for medication to modify location effects differently by timepoint. In the stratified analyses of pollutant-endpoint associations, participants with ischaemic heart disease who did not use medication showed significant associations of pulse wave velocity with black carbon, NO_2_, and ultrafine particles, while those who used medication did not show such associations; however, this was not recorded with augmentation index (figure 8C).

We checked the effect of smoking history measured by the pack-years of smoking (number of 20 cigarette-packs smoked per day multiplied by years of smoking) on the changes of health outcomes between the two sites as an independent variable. We found no significant effects of smoking in the site analysis (p values ranging from 0·093 to 0·984), such that smoking did not modify the site effects on the walk-induced changes in health outcomes (appendix).

## Discussion

In healthy volunteers, walking for 2 h in a relatively less polluted urban park (Hyde Park) was associated with a bronchodilator response present up to 26 h, accompanied by a smaller increase in FVC sustained at 5 h, but with no changes in small airways function. By contrast, after walking in the more polluted commercial roadside (Oxford Street), there were no significant changes in FEV_1_ or FVC from baseline, suggesting that pollutant exposures suppressed the walking-induced increase in FEV_1_. Both COPD and ischaemic heart disease participants experienced the beneficial effect of walking in both locations in terms of an improvement in FEV_1_, although this was less than that recorded in healthy volunteers. This is consistent with our findings on the significant associations between FEV_1_ and pollutant concentrations in participants with COPD. There was also an increase in small airways obstruction measured as R5–20 in association with pollutants in COPD participants but not in healthy and ischaemic heart disease participants. This greater effect of walking in a commercial roadside on lung function of participants with COPD was associated with more respiratory symptoms of cough and sputum, shortness of breath, and wheeze, while participants with ischaemic heart disease and the healthy volunteers were mostly asymptomatic walking on a commercial roadside except for increased cough in individuals with ischaemic heart disease.

The findings related to the cardiovascular parameters of pulse wave velocity and augmentation index, which are measures of arterial stiffness,^22^ are also of significant interest. In healthy participants, walking in Hyde Park led to a reduction in arterial stiffness that persisted up to 26 h, a benefit that was not only lost but even reversed after walking on Oxford Street. Participants with COPD or ischaemic heart disease also exhibited a reduced pulse wave velocity after walking in Hyde Park but increased pulse wave velocity after walking on Oxford Street. All three groups showed reductions in augmentation index following Hyde Park; and this beneficial change was significantly attenuated (even reversed at a few timepoints) after the Oxford Street walk. Moreover, we noted pollutant effects on pulse wave velocity and augmentation index mostly in participants with COPD and healthy volunteers but not in participants with ischaemic heart disease. This counter-intuitive finding could be due to the protective effect of the use of medications such as statins, angiotensin-converting-enzyme inhibitors, and calcium channel blockers, which improve arterial stiffness.^23^, ^24^, ^25^ Sensitivity analyses of ischaemic heart disease participants, stratified by medication use versus no medication use, confirmed that only those who did not use medication had attenuated reductions in pulse wave velocity and augmentation index after the Oxford Street walk compared with those after Hyde Park walks. By contrast, participants with ischaemic heart disease who used medications did not show differences in pulse wave velocity and augmentation index changes between the Oxford Street and the Hyde Park walks. The stratified analyses of pollutant associations showed significant associations of pulse wave velocity, but not augmentation index, with components of pollution, supporting a protective effect of cardiovascular medication.

While there was no association of pollutants with spirometric measurements in healthy and ischaemic heart disease participants, the fall in FEV_1_ and FVC was related to changes in noise, black carbon, and ultrafine particles. These results are in agreement with our previous asthma study where the reductions in FEV_1_ and FVC were associated with ultrafine particles but not with PM_2.5_.^20^ Examination of the cardiovascular parameters also showed an association of black carbon, and ultrafine particles, and not PM_2.5_, with increases in pulse wave velocity and augmentation index in healthy and COPD participants. Overall, we found that participants with COPD were especially sensitive to black carbon and ultrafine particles in terms of both pulmonary and cardiovascular effects. Our findings are in line with the cohort analyses showing an association between traffic related air pollutants (PM_2.5_, black carbon and ultrafine particles) and cardiovascular disease and mortality.^26^, ^27^, ^28^ This supports the view that pollutants emitted from fossil fuel combustion are particularly toxic for individuals with cardiovascular or pulmonary diseases.

The effect of exposure to pollution in natural environments on the cardiovascular and respiratory systems has been reported in young fit volunteers during high-intensity or submaximal exercise such as cycling or running. Running along or near busy highways or cycling during the rush hour has been shown to cause a reduction in lung function such as FEV_1_.^29^, ^30^ On the other hand, the beneficial effects of aerobic exercise on the cardiovascular system has been documented with submaximal exercise causing a reduction in arterial distensibility and thus arterial stiffness in normal volunteers.^31^ Our study extends the respiratory and cardiovascular benefits of normal walking exercise in older healthy adults and in patients with chronic stable respiratory and cardiovascular diseases. It also shows how there are detrimental effects of road traffic pollution on these benefits of short-term walking, and implicates most of the components of the pollutants being associated with the changes in lung function and indices of arterial stiffness.

There are some limitations to this study. Because we did not include a resting control group, it would not be possible to be certain that walking contributed to the changes in lung function or arterial stiffness. Another consideration is the potential contribution of diurnal variation on the spirometric measurements.^32^ However, because the first baseline measurements were made at 11 00 h, which is 1 h before the peak increase in FEV_1_ that has been reported,^32^ the increases in FEV_1_ measured after the first hour in Hyde Park in healthy participants were not related to diurnal variation but most likely induced by the walk. Second, the gender imbalance between the groups might have had an effect on the responses, but no significant differences in endpoints were recorded between sexes. Finally, the effect of medications taken by participants with either ischaemic heart disease or COPD on the cardiovascular and pulmonary responses to walking cannot be excluded; however, a study in which therapies were discontinued would not be ethically justifiable. Indeed, our data suggest that cardioprotective drugs taken by participants with ischaemic heart disease are beneficial when walking in polluted areas.

Our short-term study is unlikely to inform on the long-term benefits of exercise in relation to pollution. Moderate physical activity might protect against the adverse effects of air pollution on arterial stiffness,^33^ providing support that regular walking could lead to restoration of the protective pulmonary and cardiovascular benefits of walking. Data from other studies suggest that air pollution risks do not overcome the benefits of active walking in urban areas using all-cause mortality outcome.^34^, ^35^ Furthermore, the use of mortality as the main outcome has its limitation, because pulmonary and cardiovascular parameters that are indicative of development of potential respiratory and cardiovascular conditions caused by exposure to pollutants would probably be more relevant, especially in participants with chronic pulmonary and cardiovascular diseases who are more susceptible to the toxic effects of pollutants.^2^, ^8^, ^36^ The long-term effects of regular walking in polluted environments in these chronic conditions need to be established.

We have noted that adults free of chronic cardiopulmonary diseases lose the benefits of walking on pulmonary and cardiovascular function in a polluted environment. In participants with COPD and ischaemic heart disease exposed to traffic pollutants, the pulmonary benefit from walking seem to be lost too, but the improvement in arterial stiffness caused by walking is relatively well preserved in ischaemic heart disease participants, likely due to concomitant routine medication use. Given that walking at a normal pace provides these cardiovascular and respiratory benefits, our results support the advice that healthy participants and patients with COPD and ischaemic heart disease should avoid highly polluted areas to walk in, if the benefits of walking exercise are to be fully reaped. Walking is a preferred daily natural and essential activity highly suited for older adults and for people with chronic lung and heart disease, and patients with chronic heart disease should preferably be on cardioprotective medications. Although further studies are needed, particularly looking at the effect of regular walking in polluted environments, the current data are sufficiently compelling to advise older adults and chronic pulmonary and cardiac participants to avoid walking in highly polluted environments such as city streets with high traffic density. Exercise such as walking should be done in urban green space areas away from high density traffic or in indoor facilities with effective air filtration if located near polluted streets. Finally, it is important to impose policies and measures that can reduce traffic pollution so that every individual can enjoy the health benefits of physical activity.

This online publication has been corrected. The corrected version first appeared at thelancet.com on January 25, 2018
