# Department of Error

**DOI:** 10.1016/S0140-6736(18)30099-0

**Published:** 2018-01-27

*Sinharay R, Gong J, Barratt B, et al. Respiratory and cardiovascular responses to walking down a traffic-polluted road compared with walking in a traffic-free area in participants older than 60 years with chronic lung or heart disease and age-matched healthy controls: a randomised, crossover study.* Lancet *2017; **391:** 339–49—*In this Article (published online first on Dec 5, 2017), the corresponding author has been corrected, the middle initial for Frank Kelly has been added, the role of the funding source has been updated, and author initials have been updated throughout. These corrections have been made to the online version as of Jan 25, 2018, and the printed Article is correct.

