# Supplementary material for: Respiratory and cardiovascular responses to walking down a traffic-polluted road compared with walking in a traffic-free area in participants aged 60 years and older with chronic lung or heart disease and age-matched healthy controls: a randomised, crossover study
Source: Lancet. 2018 Jan 27;391(10118):339–49. doi: 10.1016/S0140-6736(17)32643-0 (PMC5803182; doi:10.1016/S0140-6736(17)32643-0)
Supplement: Supplementary appendix [file mmc1.pdf]

THE LANCET

Supplementary appendix

This appendix formed part of the original submission and has been peer reviewed. We post it as supplied by the authors.

Supplement to: Sinharay R, Gong J, Barratt B, et al. Respiratory and cardiovascular responses to walking down a traffic-polluted road compared with walking in a traffic-free area in participants older than 60 years with chronic lung or heart disease and age-matched healthy controls: a randomised, crossover study. *Lancet* 2017; published online Dec 5. http://dx.doi.org/10.1016/S0140-6736(17)32643-0.

Supplementary Materials

Cardio-pulmonary responses to walking on a traffic-polluted roadside in healthy, pulmonary and cardiac participants

Rudy Sinharay[1,2,#], Jicheng Gong[3,4,#], Benjamin Barratt[5], Pamela Ohman-Strickland[6], Sabine Ernst[2], Frank Kelly[5], Junfeng (Jim) Zhang[4,3], Peter Collins[1,2], Paul Cullinan[1,2,*], Kian Fan Chung[1,2,*]

[1]National Heart & Lung Institute & MRC-PHE Centre for Environment and Health, Imperial College, London SW3, UK; [2] NIHR Biomedical Research Unit, Royal Brompton & Harefield NHS Trust, London, UK;[3]College of Environmental Science and Engineering and BIC-ESAT, Peking University, Beijing, China ; [4] Nicholas School of Environment & Duke Global Health Institute, Duke University, Durham, USA, and Duke Kunshan University, Kunshan, China; [5]MRC-PHE Centre for Environment and Health, King's College London, London, UK; [6]Rutgers School of Public Health, Rutgers, The State University of New Jersey, New Jersey, USA

<u>Supplementary Materials</u>:

Supplementary Tables S1, S2, S3

Supplementary Figures S1, S2, S3

Supplementary Materials

Table S1. Estimated percent changes (and 95% confidence interval) in the health outcomes from the baseline and at various hours after the start of the walk (time at 0hr) in Oxford Street or in Hyde Park for healthy, COPD and IHD participants.

Health outcomes	Time (hours)	Healthy				COPD				IHD			
		Oxford Street		Hyde Park		Oxford Street		Hyde Park		Oxford Street		Hyde Park	
		Estimate	95% CI	Estimate	95% CI	Estimate	95% CI	Estimate	95% CI	Estimate	95% CI	Estimate	95% CI
FEV_1 (% predicted)	1	0.66	(-1.77, 3.10)	3.08	(0.51, 5.66)	-3.48	(-7.64, 0.68)	3.50	(-0.75, 7.76)	0.44	(-2.23, 3.10)	0.36	(-2.24, 2.97)
	2	-0.04	(-2.47, 2.40)	2.17	(-0.40, 4.75)	-2.19	(-6.36, 1.97)	-0.38	(-4.64, 3.87)	2.33	(-0.34, 4.99)	0.52	(-2.06, 3.11)
	3	3.41	(0.98, 5.84)	5.63	(3.05, 8.20)	5.08	(0.92, 9.24)	9.21	(4.96, 13.47)	3.01	(0.34, 5.67)	4.45	(1.87, 7.04)
	5	1.91	(-0.53, 4.34)	7.64	(5.07, 10.22)	3.31	(-0.86, 7.47)	4.60	(0.35, 8.85)	3.16	(0.50, 5.83)	4.11	(1.52, 6.69)
	6	1.85	(-0.58, 4.29)	7.42	(4.85, 10.00)	3.22	(-0.95, 7.38)	4.41	(0.16, 8.67)	4.40	(1.74, 7.07)	4.62	(2.04, 7.21)
	26	0.12	(-2.32, 2.55)	3.55	(0.98, 6.13)	3.75	(-0.41, 7.91)	5.16	(0.91, 9.41)	1.37	(-1.30, 4.03)	2.13	(-0.46, 4.71)
FVC (% predicted)	1	0.10	(-2.54, 2.74)	1.21	(-1.45, 3.87)	-1.79	(-4.94, 1.37)	3.98	(0.86, 7.10)	-0.09	(-3.10, 2.92)	3.61	(0.74, 6.48)
	2	-0.91	(-3.55, 1.73)	0.48	(-2.18, 3.14)	-3.66	(-6.81, -0.50)	1.69	(-1.43, 4.80)	3.03	(0.02, 6.04)	5.18	(2.34, 8.03)
	3	0.72	(-1.92, 3.36)	3.07	(0.41, 5.73)	2.21	(-0.95, 4.79)	6.04	(2.93, 9.16)	3.42	(0.41, 6.63)	5.59	(2.74, 8.43)
	5	0.55	(-2.09, 3.19)	4.36	(1.70, 7.02)	1.63	(-1.52, 4.79)	4.82	(1.70, 7.93)	1.86	(-1.15, 4.87)	5.78	(2.93, 8.62)
	6	0.31	(-2.33, 2.95)	2.38	(-0.28, 5.05)	1.43	(-1.72, 4.59)	3.09	(-0.03, 6.21)	4.32	(1.31, 7.33)	5.97	(3.12, 8.81)
	26	-0.31	(-2.95,	-1.08	(-3.75,	1.86	(-1.29,	3.18	(0.06,	0.15	(-2.86,	4.80	(1.96,

			2.33)		1.58)		5.02)		6.30)		3.16)		7.65)
R5	4	-1.32	(-6.58, 3.94)	-3.42	(-8.87, 2.04)	4.19	(0.43, 7.95)	-4.00	(-7.78, -0.21)	4.79	(-0.44, 10.0)	0.31	(-4.62, 5.23)
	26	-2.30	(-7.56, 2.96)	-3.14	(-8.63, 2.36)	-1.67	(-5.43, 2.09)	-6.58	(-10.4, -2.80)	2.24	(-2.99, 7.47)	-3.84	(-8.76, 1.09)
R20	4	-1.41	(-5.66, 2.84)	-2.92	(-7.22, 1.38)	1.17	(-2.70, 5.03)	-7.89	(-11.7, -4.13)	-0.50	(-5.05, 4.05)	-3.34	(-7.47, 0.79)
	26	-2.51	(-6.76, 1.74)	-3.80	(-8.10, 0.51)	-1.00	(-4.87, 2.86)	-6.97	(-10.7, -3.21)	-0.84	(-5.38, 3.71)	-2.39	(-6.52, 1.74)
R5-R20	4	4.05	(-20.1, 20.1)	-4.81	(-28.2, 14.3)	11.29	(3.10, 17.0)	1.21	(-3.25, 11.8)	17.08	(8.82, 40.8)	15.23	(-4.16, 24.2)
	26	0.86	(-21.7, 18.5)	-2.76	(-25.6, 17.3)	-6.83	(-9.91, 3.98)	-9.15	(-13.4, 1.66)	14.07	(-2.25, 29.7)	5.79	(-18.8, 10.2)
Pulse Wave Velocity (PWV)	3	-0.35	(-5.36, 4.66)	-5.31	(-10.4, -0.27)	2.12	(-2.46, 6.71)	-6.09	(-10.5, -1.67)	0.65	(-4.20, 5.49)	-7.63	(-12.1, -3.20)
	5	2.74	(-2.26, 7.75)	-2.93	(-7.96, 2.11)	3.36	(-1.22, 7.95)	-5.06	(-9.49, -0.64)	5.02	(0.17, 9.87)	-5.12	(-9.56, -0.69)
	6	3.67	(-1.34, 8.68)	-2.07	(-7.13, 2.99)	3.66	(-0.92, 8.24)	-2.52	(-6.94, 1.90)	3.42	(-1.43, 8.26)	-1.62	(-6.05, 2.81)
	26	7.17	(2.16, 12.2)	-4.39	(-9.49, 0.71)	3.75	(-0.83, 8.34)	-4.70	(-9.12, -0.28)	2.91	(-1.94, 7.75)	-5.35	(-9.78, -0.91)

Table S1. Cont'd

Health outcomes	Time (hours)	Healthy				COPD				IHD			
		Oxford Street		Hyde Park		Oxford Street		Hyde Park		Oxford Street		Hyde Park	
		Estimate	95% CI	Estimate	95% CI	Estimate	95% CI	Estimate	95% CI	Estimate	95% CI	Estimate	95% CI
Augmentation Index (AI)	3	-4.62	(-11.6, 2.36)	-24.27	(-31.0, -17.5)	-16.16	(-22.9, -9.47)	-24.64	(-31.0, -18.3)	-8.64	(-15.9, -1.35)	-19.75	(-26.5, -13.0)
	5	-1.77	(-8.75, 5.21)	-16.87	(-23.6, -10.1)	-4.80	(-11.5, 1.89)	-15.40	(-21.7, -9.07)	-6.69	(-14.0, 0.60)	-12.70	(-19.4, -5.99)
	6	-2.91	(-9.89, 4.07)	-13.90	(-20.7, -7.12)	1.02	(-5.67, 7.71)	-13.89	(-20.2, -7.55)	-2.79	(-10.1, 4.51)	-11.27	(-18.0, -4.56)
	26	3.27	(-3.71, 10.3)	-8.76	(-15.6, -1.93)	3.84	(-2.85, 10.5)	-16.74	(-23.1, -10.4)	0.57	(-6.72, 7.87)	-10.13	(-16.8, -3.41)
Systolic Blood Pressure (SBP)	3	-7.36	(-10.2, -4.47)	-6.06	(-9.07, -3.06)	-9.00	(-11.8, -6.23)	-8.72	(-11.6, -5.86)	-8.27	(-11.5, -5.03)	-10.55	(-13.6, -7.53)
	5	-1.17	(-4.06, 1.71)	-1.00	(-4.00, 2.01)	-4.37	(-7.14, -1.61)	-0.67	(-3.53, 2.19)	-1.42	(-4.66, 1.81)	-4.55	(-7.57, -1.54)
	6	3.37	(0.48, 6.26)	2.84	(-0.18, 5.86)	0.16	(-2.60, 2.93)	2.88	(0.02, 5.74)	2.14	(-1.09, 5.38)	0.10	(-2.92, 3.11)
	26	-3.45	(-6.33, -0.56)	-1.67	(-4.70, 1.37)	-5.09	(-7.88, -2.30)	-3.05	(-5.91, -0.19)	-1.47	(-4.70, 1.77)	-5.15	(-8.17, -2.13)
Heart rate	3	9.75	(5.83, 13.7)	12.06	(8.03, 16.1)	20.46	(16.7, 24.2)	16.16	(12.4, 19.9)	10.93	(6.35, 15.5)	10.91	(6.45, 15.4)
	5	-0.39	(-4.31, 3.53)	0.21	(-3.82, 4.24)	5.42	(1.65, 9.18)	2.56	(-1.16, 6.28)	1.92	(-2.66, 6.51)	1.41	(-3.04, 5.87)
	6	-3.01	(-6.92, 0.91)	-2.47	(-6.51, 1.58)	1.62	(-2.15, 5.39)	1.40	(-2.32, 5.12)	2.42	(-2.17, 7.00)	0.09	(-4.37, 4.55)
	26	1.52	(-2.40, 5.44)	0.42	(-3.65, 4.49)	5.63	(1.86, 9.40)	4.64	(0.92, 8.37)	2.31	(-2.27, 6.90)	3.55	(-0.91, 8.01)

Table S2. Percent change estimate, standard error (SE), and 95% confidence interval (95% CI) in FEV_1 (% predicted value) and FVC (% predicted value) , in differential frequency-dependent respiratory resistance at 5Hz and 20Hz (R5-20), in pulse wave velocity (PWV) and augmentation index (AI) per interquartile range changes in components of air pollution at the times indicated after the start of the two-hour walk in two exposure sessions for each group of healthy, COPD and IHD participants.

Health outcomes	Pollutant	Healthy				COPD				IHD			
		Estimate	SE	95% CI-lower level	95% CI-upper level	Estimate	SE	95% CI-lower level	95% CI-upper level	Estimate	SE	95% CI-lower level	95% CI-upper level
FEV_1 (% predicted)	BC	-0.42	1.64	-3.65	2.80	-3.21	2.54	-8.19	1.77	2.43	1.80	-1.09	5.96
	NO_2	-0.18	1.37	-2.87	2.50	-4.85	2.13	-9.03	-0.67	-1.03	1.22	-3.41	1.35
	Noise	-1.05	0.88	-2.78	0.68	-1.93	1.75	-5.35	1.48	-1.92	1.11	-4.10	0.27
	UFP	-0.98	1.51	-3.94	1.97	-5.28	2.60	-10.37	-0.19	1.29	1.88	-2.39	4.96
	$PM_{2.5}$	-0.65	1.07	-2.74	1.44	-4.19	1.84	-7.80	-0.58	0.43	1.24	-1.99	2.85
	PM_{10}	0.05	0.93	-1.77	1.87	-2.61	1.55	-5.65	0.43	0.42	1.10	-1.73	2.58
FVC (% predicted)	BC	0.38	1.86	-3.27	4.03	-2.60	2.0	-6.53	1.34	0.46	2.12	-3.69	4.61
	NO_2	-0.36	1.54	-3.38	2.66	-4.99	1.67	-8.27	-1.70	-1.59	1.42	-4.38	1.19
	Noise	0.24	1.00	-1.71	2.20	-4.39	1.39	-7.12	-1.65	-1.81	1.33	-4.41	0.79
	UFP	-1.90	1.72	-5.27	1.48	-4.87	2.07	-8.93	-0.81	-1.47	2.23	-5.84	2.91
	$PM_{2.5}$	-0.16	1.22	-2.55	2.23	-3.60	1.47	-6.48	-0.72	-1.23	1.47	-4.10	1.65
	PM_{10}	0.23	1.08	-1.88	2.34	-3.41	1.24	-5.84	-0.98	-1.31	1.31	-3.88	1.26
R5-R20	BC	14.66	9.50	-3.96	33.27	22.63	8.28	6.39	38.86	16.54	10.9	-4.82	37.90
	NO_2	6.07	7.61	-8.84	20.99	26.37	6.83	12.98	39.75	8.38	7.22	-5.97	22.73
	Noise	5.71	4.52	-3.16	14.57	8.49	6.07	-3.41	20.39	-4.23	5.55	-15.30	6.83
	UFP	11.04	8.39	-5.40	27.48	24.26	8.08	8.41	40.10	15.00	11.0	-6.56	36.56
	$PM_{2.5}$	-3.75	6.25	-16.01	8.50	12.79	5.82	1.38	24.21	2.91	8.87	-14.47	20.30

	PM_{10}	0.47	5.34	-10.00	10.95	10.24	4.94	0.55	19.93	4.39	7.84	-10.99	19.76
Pulse Wave Velocity (PWV)	BC	9.80	3.36	3.22	16.38	7.36	2.67	2.14	12.59	3.70	3.29	-2.76	10.15
	NO_2	2.66	2.54	-2.31	7.63	3.28	2.02	-0.67	7.24	1.81	2.01	-2.13	5.74
	Noise	4.85	1.72	1.48	8.22	1.51	1.78	-1.98	5.01	4.12	1.92	0.37	7.88
	UFP	11.37	2.89	5.71	17.04	7.77	2.59	2.70	12.84	5.20	3.23	-1.14	11.53
	$PM_{2.5}$	-0.09	2.15	-4.30	4.13	3.00	1.88	-0.68	6.69	0.91	2.19	-3.38	5.20
	PM_{10}	2.20	1.87	-1.47	5.88	2.13	1.59	-0.98	5.25	2.68	1.92	-1.09	6.45
Augmentation Index (AI)	BC	18.39	4.50	9.58	27.21	10.50	4.01	2.63	18.36	4.23	4.76	-5.10	13.57
	NO_2	15.00	3.48	8.18	21.82	12.48	3.11	6.39	18.57	1.54	2.99	-4.33	7.40
	Noise	0.12	2.23	-4.25	4.49	1.54	2.51	-3.37	6.46	0.61	2.61	-4.51	5.72
	UFP	16.18	3.95	8.45	23.91	9.97	3.95	2.22	17.72	7.89	4.76	-1.45	17.22
	$PM_{2.5}$	1.27	2.80	-4.21	6.75	4.36	2.79	-1.11	9.83	5.18	3.15	-1.00	11.36
	PM_{10}	1.33	2.40	-3.37	6.04	1.70	2.36	-2.93	6.32	4.17	2.79	-1.30	9.63

Table S3. Estimated percent changes (and 95% confidence interval) in the health outcomes from the baseline and at various hours after the start of the walk (time at 0hr) in Oxford Street or in Hyde Park for healthy, COPD and IHD participants after controlling for the pack-year for the sensitivity analysis of smoking status.

Health outcomes	Time (hours)	Healthy				COPD				IHD			
		Oxford Street		Hyde Park		Oxford Street		Hyde Park		Oxford Street		Hyde Park	
		Estimate	95% CI	Estimate	95% CI	Estimate	95% CI	Estimate	95% CI	Estimate	95% CI	Estimate	95% CI
FEV_1 (% predicted)	1	0.39	(-2.11, 2.89)	2.79	(0.14, 5.44)	-2.74	(-7.15, 1.66)	4.26	(-0.25, 8.77)	0.26	(-2.47, 2.99)	0.03	(-2.62, 2.68)
	2	-0.31	(-2.81, 2.19)	1.88	(-0.77, 4.52)	-1.46	(-5.86, 2.95)	0.37	(-4.13, 4.88)	2.14	(-0.59, 4.88)	0.06	(-2.60, 2.71)
	3	3.14	(0.64, 5.64)	5.33	(2.68, 7.98)	5.81	(1.41, 10.22)	9.97	(5.47, 14.48)	2.81	(0.08, 5.55)	4.27	(1.62, 6.92)
	5	1.63	(-0.87, 4.14)	7.35	(4.70, 9.99)	4.04	(-0.37, 8.45)	5.36	(0.85, 9.86)	3.04	(0.30, 5.77)	3.86	(1.21, 6.51)
	6	1.58	(-0.92, 4.08)	7.13	(4.48, 9.77)	3.95	(-0.45, 8.36)	5.17	(0.67, 9.68)	4.38	(1.64, 7.11)	4.48	(1.83, 7.13)
	26	-0.15	(-2.66, 2.35)	3.26	(0.61, 5.91)	4.48	(0.08, 8.89)	5.92	(1.41, 10.42)	1.15	(-1.58, 3.89)	2.00	(-0.65, 4.65)
FVC (% predicted)	1	-0.08	(-2.81, 2.64)	1.04	(-1.71, 3.78)	-1.43	(-4.80, 1.93)	4.33	(1.00, 7.66)	-0.21	(-3.30, 2.89)	3.41	(0.48, 6.33)
	2	-1.09	(-3.82, 1.63)	0.31	(-2.44, 3.05)	-3.30	(-6.67, 0.06)	2.04	(-1.29, 5.36)	3.10	(0.01, 6.19)	4.85	(1.93, 7.77)
	3	0.54	(-2.18, 3.26)	2.89	(0.15, 5.64)	2.56	(-0.80, 5.92)	6.39	(3.07, 9.72)	3.44	(0.35, 6.53)	5.66	(2.73, 8.58)
	5	0.37	(-2.35, 3.09)	4.19	(1.44, 6.93)	1.98	(-1.38, 5.35)	5.17	(1.84, 8.49)	1.80	(-1.29, 4.90)	5.82	(2.90, 8.74)

	6	0.13	(-2.59, 2.85)	2.21	(-0.53, 4.95)	1.78	(-1.58, 5.15)	3.44	(0.12, 6.77)	4.49	(1.40, 7.59)	6.10	(3.18, 9.02)
	26	-0.49	(-3.22, 2.23)	-1.26	(-4.00, 1.49)	2.21	(-1.15, 5.58)	3.53	(0.20, 6.85)	-0.14	(-3.24, 2.95)	4.74	(1.82, 7.66)
R5-R20	4	2.74	(-18.0, 23.5)	-4.01	(-26.0, 18.0)	8.73	(1.26, 16.2)	2.84	(-5.28, 11.0)	28.49	(12.1, 44.9)	10.43	(-4.13, 25.0)
	26	1.18	(-19.6, 21.9)	-1.23	(-23.4, 20.9)	-4.28	(-11.8, 3.19)	-7.30	(-15.4, 0.82)	16.76	(0.39, 33.1)	-5.27	(-20.1, 9.60)
Pulse Wave Velocity (PWV)	3	-1.30	(-6.42, 3.81)	-6.24	(-11.4, -1.09)	3.51	(-1.33, 8.35)	-4.78	(-9.45, -0.12)	0.97	(-3.97, 5.91)	-8.04	(-12.6, -3.52)
	5	1.79	(-3.33, 6.91)	-3.86	(-9.01, 1.29)	4.75	(-0.09, 9.59)	-3.76	(-8.42, 0.91)	4.92	(-0.02, 9.86)	-5.66	(-10.2, -1.14)
	6	2.72	(-2.40, 7.83)	-3.00	(-8.17, 2.17)	5.05	(0.21, 9.89)	-1.21	(-5.88, 3.45)	3.57	(-1.38, 8.51)	-2.00	(-6.52, 2.52)
	26	6.21	(1.10, 11.3)	-5.32	(-10.5, -0.11)	5.14	(0.30, 9.98)	-3.39	(-8.06, 1.27)	2.81	(-2.13, 7.75)	-5.86	(-10.4, -1.34)
Augmentation Index (AI)	3	-4.65	(-11.9, 2.56)	-24.35	(-31.3, -17.4)	-16.15	(-23.3, -8.97)	-24.64	(-31.4, -17.9)	-8.93	(-16.4, -1.44)	-19.77	(-26.7, -12.9)
	5	-1.80	(-9.02, 5.41)	-16.95	(-23.9, -9.97)	-4.78	(-12.0, 2.39)	-15.40	(-22.2, -8.61)	-7.82	(-15.3, -0.32)	-12.82	(-19.7, -5.92)
	6	-2.94	(-10.2, 4.27)	-13.98	(-21.0, -6.97)	1.04	(-6.14, 8.21)	-13.89	(-20.7, -7.10)	-3.91	(-11.4, 3.58)	-11.55	(-18.4, -4.65)
	26	3.23	(-3.98, 10.5)	-8.84	(-15.9, -1.79)	3.85	(-3.32, 11.0)	-16.73	(-23.5, -9.95)	0.11	(-7.38, 7.61)	-10.37	(-17.3, -3.47)

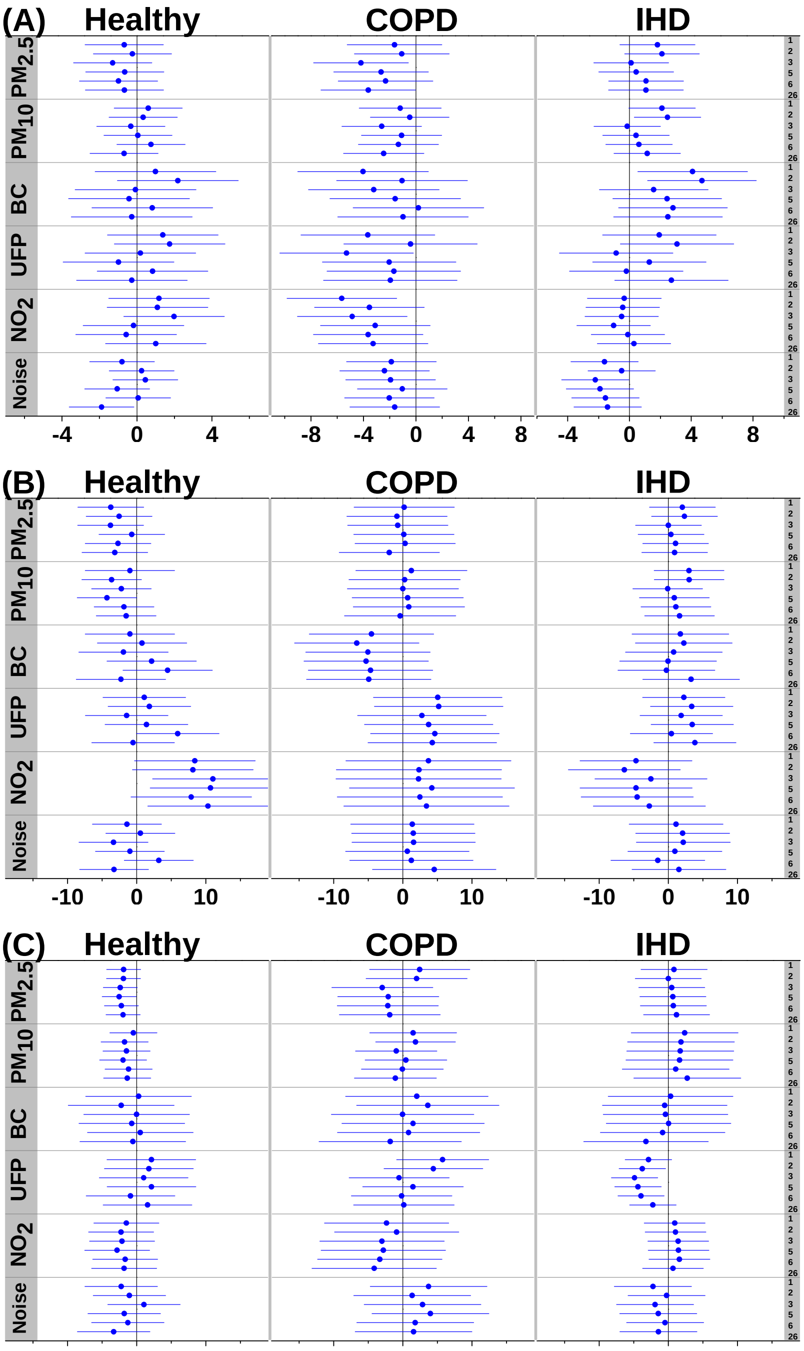

Percent Change in Outcome Associated with IQR Increase in Pollution Concentration

Figure S1. Percent changes (and 95% confidence interval) in FEV_1 (% predicted value) per interquartile range changes in components of air pollution at the times indicated after the start of the two-hour walk in two exposure sessions for each group of healthy, COPD and IHD participants at both sites (A), Oxford Street (B), and Hype Park (C). IQR of air pollution for both sites (A): $PM_{2.5}$: 14.94 μg/m^3; PM_{10}: 14.47 μg/m^3; BC: 9.2 μg/m^3; UFP: 19,854/cm^3; NO_2: 64.9 ppb; Noise: 3.97 dB. IQR of air pollution for Oxford Street (B): $PM_{2.5}$: 12.73 μg/m^3; PM_{10}: 14.86 μg/m^3; BC: 3.94 μg/m^3; UFP: 9974/cm^3; NO_2: 63.5 ppb; Noise: 3.00 dB. IQR of air pollution for Hyde Park (C): $PM_{2.5}$: 7.59 μg/m^3; PM_{10}: 11.59 μg/m^3; BC: 1.43 μg/m^3; UFP: 2585/cm^3; NO_2: 9.8 ppb; Noise: 5.68 dB.

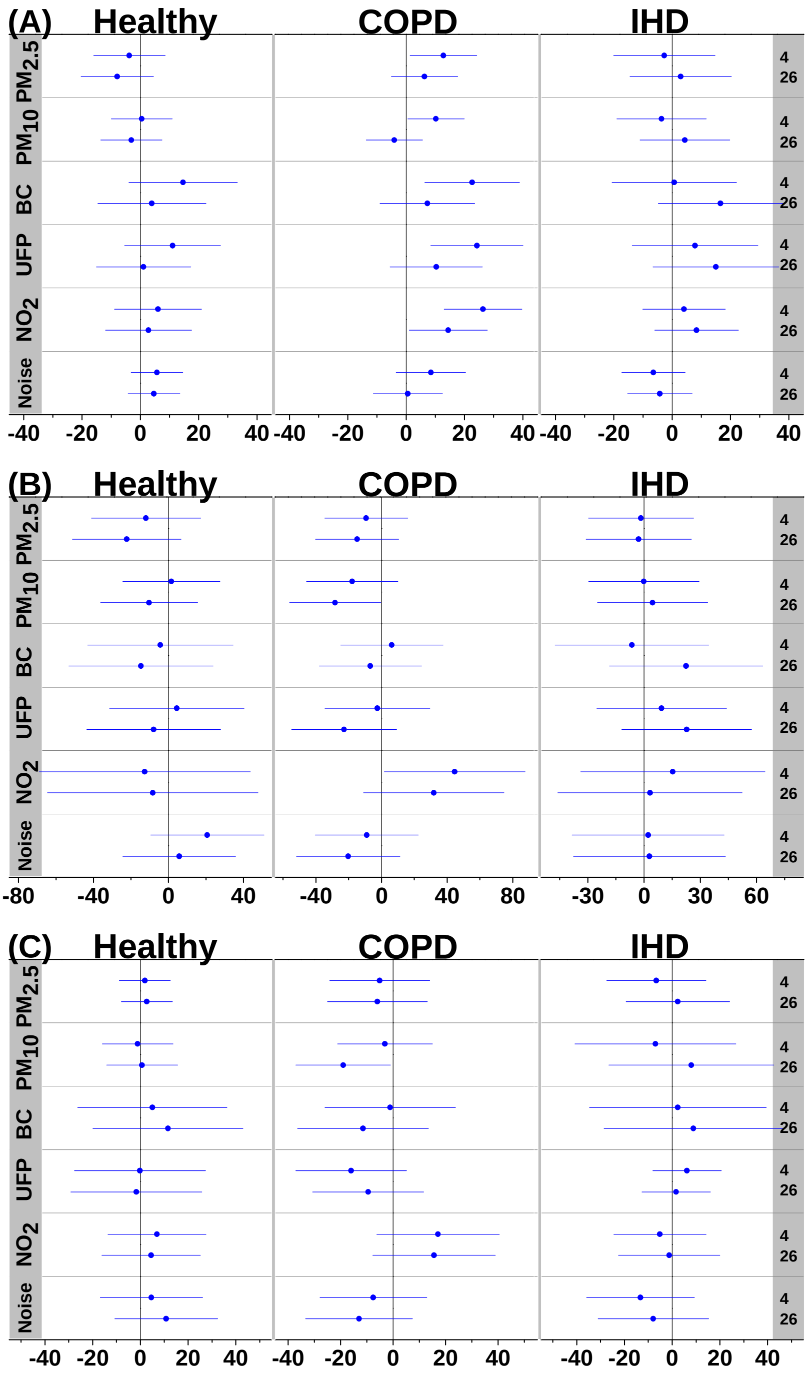

Percent Change in Outcome Associated with IQR Increase in Pollution Concentration

Figure S2. Percent changes (and 95% confidence interval) in R5-R20 per interquartile range changes in components of air pollution at the times indicated after the start of the two-hour walk in two exposure sessions for each group of healthy, COPD and IHD participants at both sites (A), Oxford Street (B), and Hype Park (C). IQR of air pollution for both sites (A): $PM_{2.5}$: 14.94 µg/m^3; PM_{10}: 14.47 µg/m^3; BC: 9.2 µg/m^3; UFP: 19,854/cm^3; NO_2: 64.9 ppb; Noise: 3.97 dB. IQR of air pollution for Oxford Street (B): $PM_{2.5}$: 12.73 µg/m^3; PM_{10}: 14.86 µg/m^3; BC: 3.94 µg/m^3; UFP: 9974/cm^3; NO_2: 63.5 ppb; Noise: 3.00 dB. IQR of air pollution for Hyde Park (C): $PM_{2.5}$: 7.59 µg/m^3; PM_{10}: 11.59 µg/m^3; BC: 1.43 µg/m^3; UFP: 2585/cm^3; NO_2: 9.8 ppb; Noise: 5.68 dB.

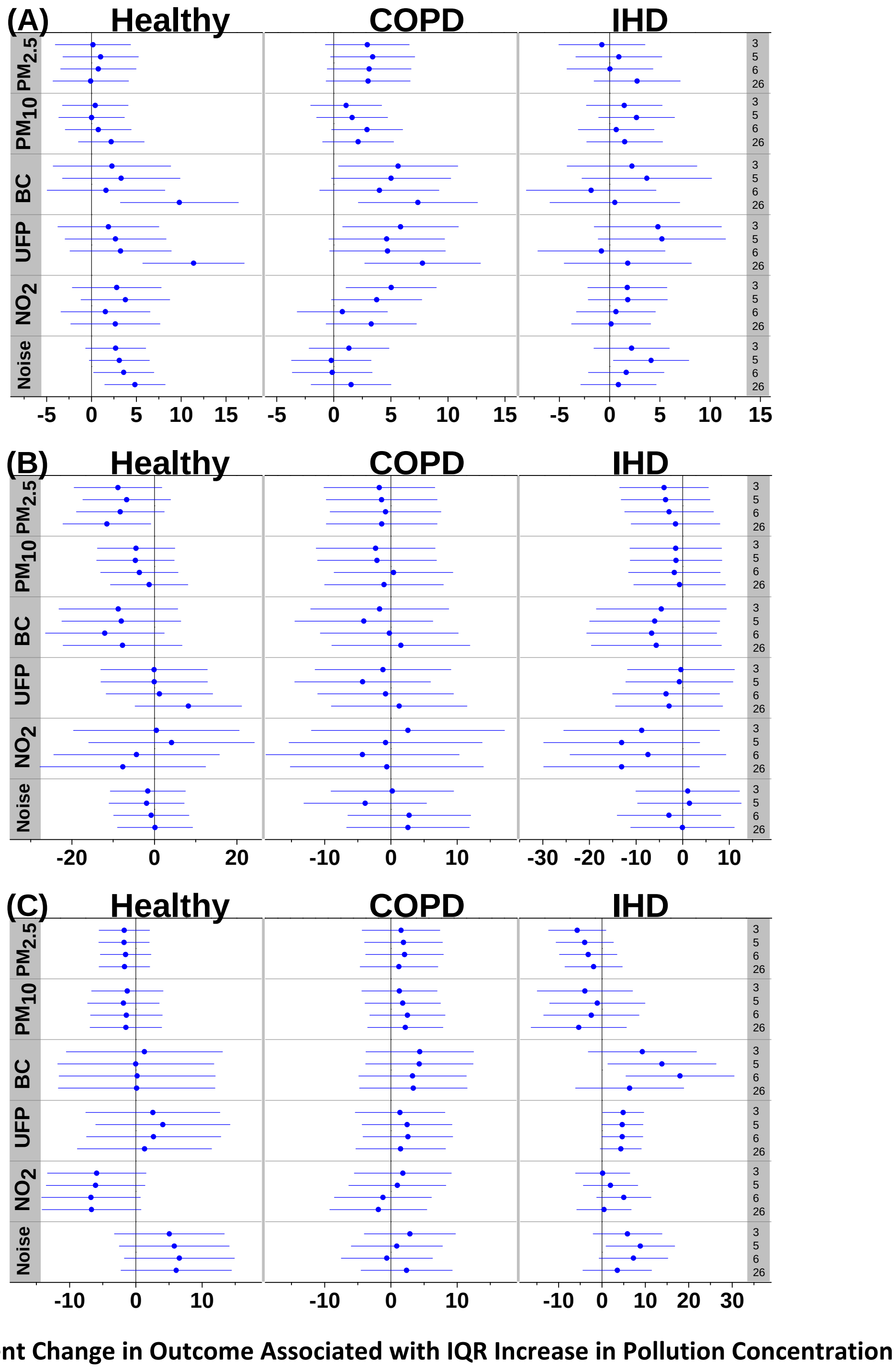

Figure S3. Percent changes (and 95% confidence interval) in PWV per interquartile range changes in components of air pollution at the times indicated after the start of the two-hour walk in two exposure sessions for each group of healthy, COPD and IHD participants at both sites (A), Oxford Street (B), and Hype Park (C). IQR of air pollution for both sites (A): $PM_{2.5}$: 14.94 μg/m^3; PM_{10}: 14.47 μg/m^3; BC: 9.2 μg/m^3; UFP: 19,854/cm^3; NO_2: 64.9 ppb; Noise: 3.97 dB. IQR of air pollution for Oxford Street (B): $PM_{2.5}$: 12.73 μg/m^3; PM_{10}: 14.86 μg/m^3; BC: 3.94 μg/m^3; UFP: 9974/cm^3; NO_2: 63.5 ppb; Noise: 3.00 dB. IQR of air pollution for Hyde Park (C): $PM_{2.5}$: 7.59 μg/m^3; PM_{10}: 11.59 μg/m^3; BC: 1.43 μg/m^3; UFP: 2585/cm^3; NO_2: 9.8 ppb; Noise: 5.68 dB.
